# Invasive Fishes Generate Biogeochemical Hotspots in a Nutrient-Limited System

**DOI:** 10.1371/journal.pone.0054093

**Published:** 2013-01-16

**Authors:** Krista A. Capps, Alexander S. Flecker

**Affiliations:** 1 Department of Ecology and Evolutionary Biology, Cornell University, Ithaca, New York, United States of America; 2 Sustainability Solutions Initiative, University of Maine, Orono, Maine, United States of America; Consiglio Nazionale delle Ricerche (CNR), Italy

## Abstract

Fishes can play important functional roles in the nutrient dynamics of freshwater systems. Aggregating fishes have the potential to generate areas of increased biogeochemical activity, or hotspots, in streams and rivers. Many of the studies documenting the functional role of fishes in nutrient dynamics have focused on native fish species; however, introduced fishes may restructure nutrient storage and cycling freshwater systems as they can attain high population densities in novel environments. The purpose of this study was to examine the impact of a non-native catfish (Loricariidae: *Pterygoplichthys*) on nitrogen and phosphorus remineralization and estimate whether large aggregations of these fish generate measurable biogeochemical hotspots within nutrient-limited ecosystems. Loricariids formed large aggregations during daylight hours and dispersed throughout the stream during evening hours to graze benthic habitats. Excretion rates of phosphorus were twice as great during nighttime hours when fishes were actively feeding; however, there was no diel pattern in nitrogen excretion rates. Our results indicate that spatially heterogeneous aggregations of loricariids can significantly elevate dissolved nutrient concentrations via excretion relative to ambient nitrogen and phosphorus concentrations during daylight hours, creating biogeochemical hotspots and potentially altering nutrient dynamics in invaded systems.

## Introduction

Mobile organisms can generate areas of enhanced nutrient recycling rates, or biogeochemical hotspots, that may influence primary productivity in both terrestrial and aquatic ecosystems [1]–[3]. McClain et al. [2] defined biogeochemical hotspots as small areas within a landscape matrix that show comparably high reaction rates relative to the surrounding areas. For organisms to generate biogeochemical hotspots within an ecosystem, their population densities must vary through space and/or time and the contribution of the species to nutrient remineralization rates must be significant relative to ecosystem demand [3]. Therefore, spatially or temporally heterogeneous aggregations of organisms can potentially generate hotspots of biogeochemical activity that influence patterns of nutrient remineralization and alter ecosystem nutrient dynamics.

In aquatic ecosystems, consumer-driven nutrient remineralization can be significant relative to ecosystem nutrient demand and can enhance periphyton biomass and productivity [4]–[6]. Fishes can play important roles in nutrient dynamics in freshwater ecosystems through nutrient sequestration and nutrient remineralization [3], [7], [8] and this may influence primary producer biomass and productivity [9]. For example in a study examining the influence of a native fish assemblage on nitrogen (N) and phosphorus (P) cycling in a tropical river, McIntyre et al. [3] found that the aggregate excretion of fishes was sufficient to turn over the entire ambient pool of N in the water column, the nutrient limiting primary productivity, in less than 0.3km. Though previous investigations have linked ecosystem processes such as biogeochemical cycling with native fish assemblages [3], [10]–[12], few studies have demonstrated how the effects of fish invasion alter these processes [13], [14], [15].

Armored catfishes (Loricariidae) are native to Central and South America [16], [17], and have been introduced to tropical and subtropical freshwater ecosystems throughout the globe [16], [18]–[26]. In invaded ecosystems, loricariids attain high population densities and they are thought to compete with native organisms for food resources and space [27], [28]. Natural resource managers have noted that populations of non-native loricariids create large aggregations [29]; however, the potential influence for this behavior to alter nutrient concentrations in space and time has not been explored. The purpose of this study was to document the diel changes in behavior of non-native populations of armored catfish and investigate the potential of these fishes to generate biogeochemical hotspots, or localized areas of increased nutrient concentrations, in invaded river systems. We predicted that nutrient remineralization by loricariids would represent a substantial flux of N and P. Moreover, we posited daytime aggregations of loricariids would generate biogeochemical hotspots, resulting in discontinuities of stream nutrient availability in space and time.

## Methods

### Study Site

The field work for this study was conducted in the Chacamax River (N17°29′047′′ W91°58′430′′) in Chiapas, Mexico during the dry season months of March-May 2008–2010. The benthos in the study reach was characterized by large cobble and gravel substrate. During the study, stream discharge averaged ∼1,600 L s^−1^ and water temperature in the river ranged from 21 to 28°C. Ambient nutrient concentrations, collected from sites without aggregations of armored catfish, in the study reaches were low to moderate (average values: NH_4_^+^-N, 10 µg L^−1^; NO_3_^−^-N, 353 µg L^−1^; total dissolved nitrogen, 387 µg L^−1^; soluble reactive phosphorus, <2 µg L^−1^; total dissolved phosphorus, 3 µg L^−1^). Nutrient diffusing substrates, constructed after methods outlined in Capps et al. [30], indicated primary producers in the stream were limited by P (Figure S1). Loricariid density was 2.3±3.4 m^−2^ (mean ± SD) and areal biomass was 225±45 g m^−2^ (mean ± SD), two orders of magnitude greater than the native fish biomass in the study reach in 2010 [31].

Non-native loricariids were first documented in the Chacamax River in 2004, and include *Pterygoplichthys pardalis* (Castelnau, 1855), *Pterygoplichthys disjunctivis* (Weber, 1991) and *Pterygoplichthys* that do not adhere to type specimens (Figure S2). The wide variations in pattern suggest the *Pterygoplichthys* population in the Chacamax may be comprised of hybrids of the two species; hence, we refer to the fish as *Pterygoplichthys*.

### Diel Patterns in Fish Behavior and Ambient Nutrient Concentrations

To describe diel patterns in *Pterygoplichthys* behavior, we counted the number of fish found in five 1 m×1 m quadrats along the edge of a 100 m reach of stream during daytime and nighttime hours. These measurements were collected to document loricariids were spreading out from their daytime aggregations to graze the entire stream bed. We counted *Pterygoplichthys* every four hours over a period of three days in March 2010. To document diel fluctuations in water chemistry, we collected duplicate water samples from the thalweg of the stream in single a run habitat for NH_4_^+^ and PO_4_^−3^ analysis every four hours on three dates in 2010.

To estimate the influence of time of day (day/night) on loricariid feeding behavior, 77 fish were harvested and weighed during daytime (22 fish; 1200–1730 hrs) or nighttime (55 fish; 1900–0400 hrs) hours, euthanized using an overdose of MS-222, and their gut contents were collected, dried, and weighed according to methods outlined in German and Bittong (2009) under IACUC protocol number: 2006–0169, Cornell University. All values were expressed as the ratio of gut content dry mass (g) to fish wet mass (g) to account for size variation in the fishes we sampled. Again, these measurements were taken to document pronounced, diel changes in loricariid behavior and their nocturnal foraging activities.

### Nutrient Remineralization and Hotspot Sampling

To determine nutrient remineralization rates of loricariids and estimate the effects of their remineralization on ambient water chemistry, we conducted fish excretion incubations and sampled water within and outside of aggregations of loricariids in the Chacamax River. We predicted that aggregations of loricariids would generate biogeochemical hotspots, evidenced by increased ambient nutrient concentrations from samples collected in the aggregations relative to samples collected outside of the aggregations. Twenty *Pterygoplichthys* (Standard length 23.5 cm ±5 cm(mean ± SD)) were collected using hand nets and immediately, individually incubated in 15L plastic tubs in 10L of filtered stream water for approximately 1 h. Ten incubations were conducted between 1200 and 1500 hrs (daytime) and 10 incubations occurred between 1900 and 2100 hours (nighttime). Two additional fish-free tubs were maintained as controls during each incubation period. Tubs were filled with 10L of filtered stream water and placed in the shade for the duration of the incubation. At the end of the incubation, we collected filtered water samples for NH_4_^+^ and total dissolved phosphorus (TDP) analysis. Fish nutrient recycling rates were estimated based on the difference in dissolved N and P concentrations between plastic tubs incubated with and without *Pterygoplichthys* [3], [8]. At the end of the incubation period, water samples were filtered through glass-fiber filters (Gelman A/E) and were either acidified and shipped to the USA for P analysis, or were analyzed in the field for NH_4_^+^. We used standard colorimetric methods to analyze TDP and soluble reactive phosphorus (SRP) samples (APHA 1998) using a Lachat QuickChem 8000 (Lachat Instruments, Loveland, Colorado). All NH_4_^+^samples were refrigerated and analyzed in the field using the flurometric methods outlined by Taylor et al. [32].

**Figure 1:**
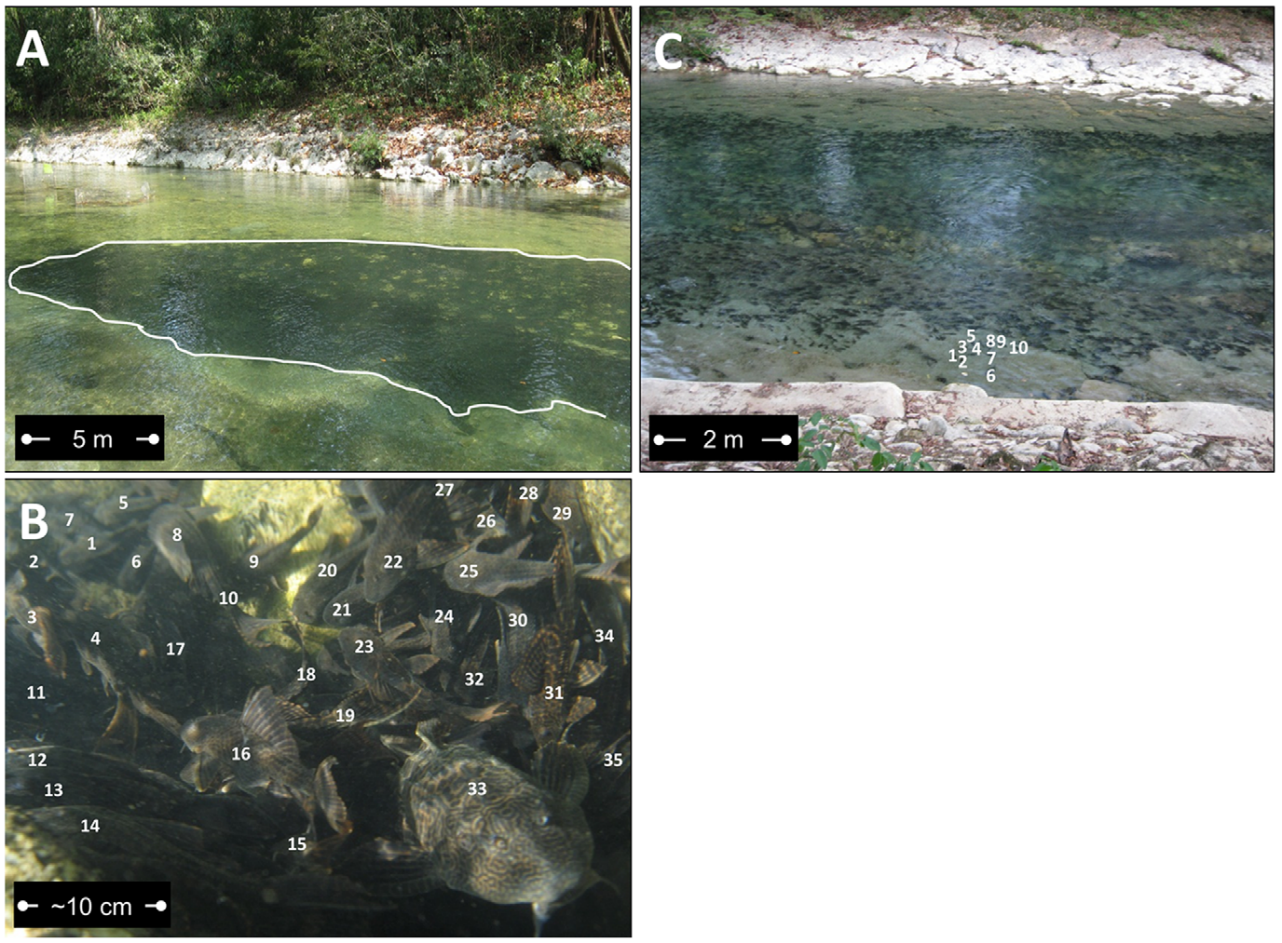
*Pterygoplichthys* in the Chacamax River (N17°29’047” W91°58’430”). (A) Daytime aggregation of loricariids. The white line outlines the aggregation boundary. (B) Underwater photo of loricariid aggregation. Individual fish are marked with white numbers (1–35). (C) Loricariids spreading out from aggregation to begin evening feeding. Each dark spot (C) is at least one *Pterygoplichthys*. A small group of fishes (1–10) have been marked with individual numbers to demonstrate fish abundance. Photo credits: K. A. Capps.

To ascertain if *Pterygoplichthys* generated hotspots of nutrient remineralization (within aggregations) relative to ambient water chemistry (outside of aggregations), we collected paired stream water samples within (i.e. within the white boundary in Figure 1A) and outside of aggregations (i.e. outside of the white boundary in Figure 1A) of loricariids in the Chacamax River in 2008 and 2010. We collected water samples from aggregations of *Pterygoplichthys* with minimum areas of 3 m^2^ with at least 50 *Pterygoplichthys* m^−2^ (Figure 1A). Paired sites were located parallel to one another along a transect extending between river banks. They were matched for similar depth (min = 0.5 m, max = 1.5 m, mean = 0.9 m) and water velocity (min = 0.04 m s^−1^, max = 0.09 m s^−1^, mean = 0.07 m s^−1^). Water samples were collected and analyzed for NH_4_^+^ and SRP using the aforementioned methods.

### Statistical Analysis

Nutrient recycling rates and gut content time comparisons (daytime/nighttime) were made using one-way ANOVAs, where time was the fixed factor. We used a mixed model to estimate the effects of fish size on nutrient recycling rates, where wet mass was considered the fixed factor and time and wet mass×time were considered random factors. Aggregation/ambient comparisons were made using a two-way ANOVA where sample site, year, and the interaction term were considered fixed factors and sample pair (aggregation/ambient) was considered a random factor. All data were log_10_ transformed to meet the assumptions of the models and analyzed using JMP 9 statistical software (SAS Institute, 2010).

## Results

### Diel Changes in Fish Behavior and Nutrient Concentrations

**Figure 2:**
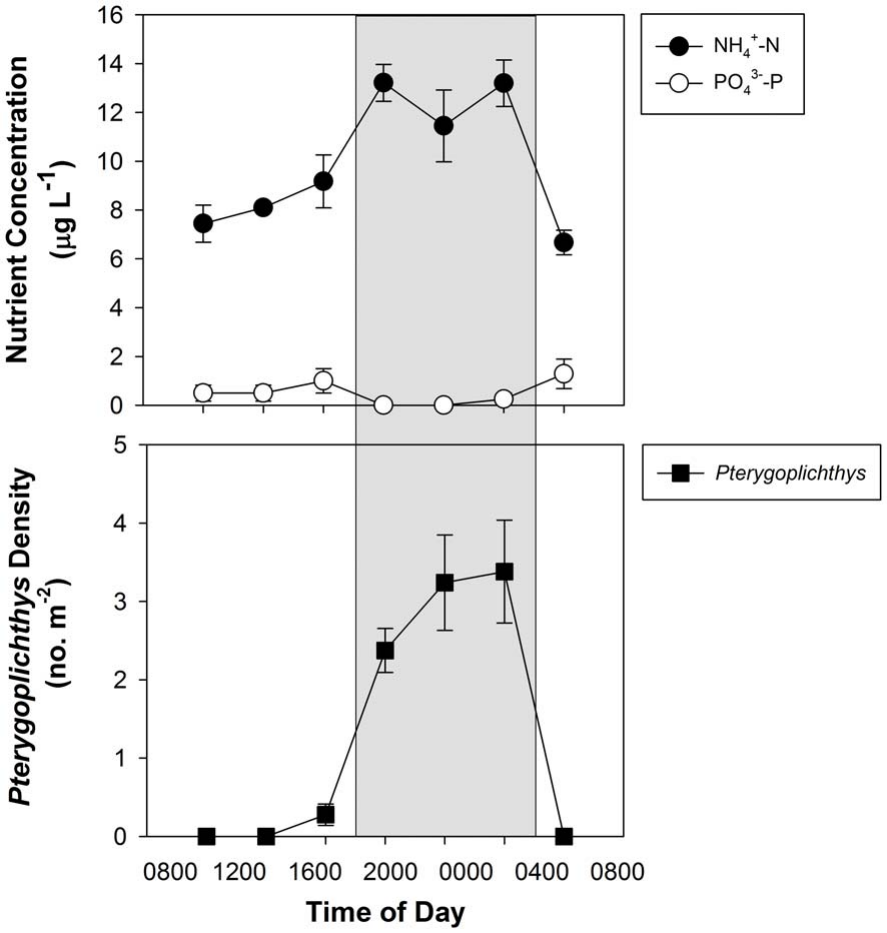
Diel changes in ambient water chemistry and *Pterygoplichthys* behavior. Data were collected in 2008 and 2010 (±1 SE). (A) NH4+-N and PO4-3-P concentrations over time; (B) number of *Pterygoplichthys* counted in 1 m^2^ quadrats near the stream bank (within 24 cm) over time. The shaded areas represent nighttime sampling hours.

Loricariids formed large aggregations in the main channel of the Chacamax River during the day, but spread out to graze the entire riverbed at night (Figures 1, 2). The aggregations occurred in same relative area each day unless there was a large discharge event that moved large, woody debris and larger cobbles. Concurrent increases in ambient NH_4_^+^ occurred at night when loricariids were broadly dispersed and actively feeding; however, there was no similar increase in ambient PO_4_^3−^ (Figure 2). Importantly, all of the PO_4_^3−^ samples were at or near detection; thus, any change in ambient levels would have been difficult to detect.

### Nutrient Recycling and Hotspot Observations

**Figure 3:**
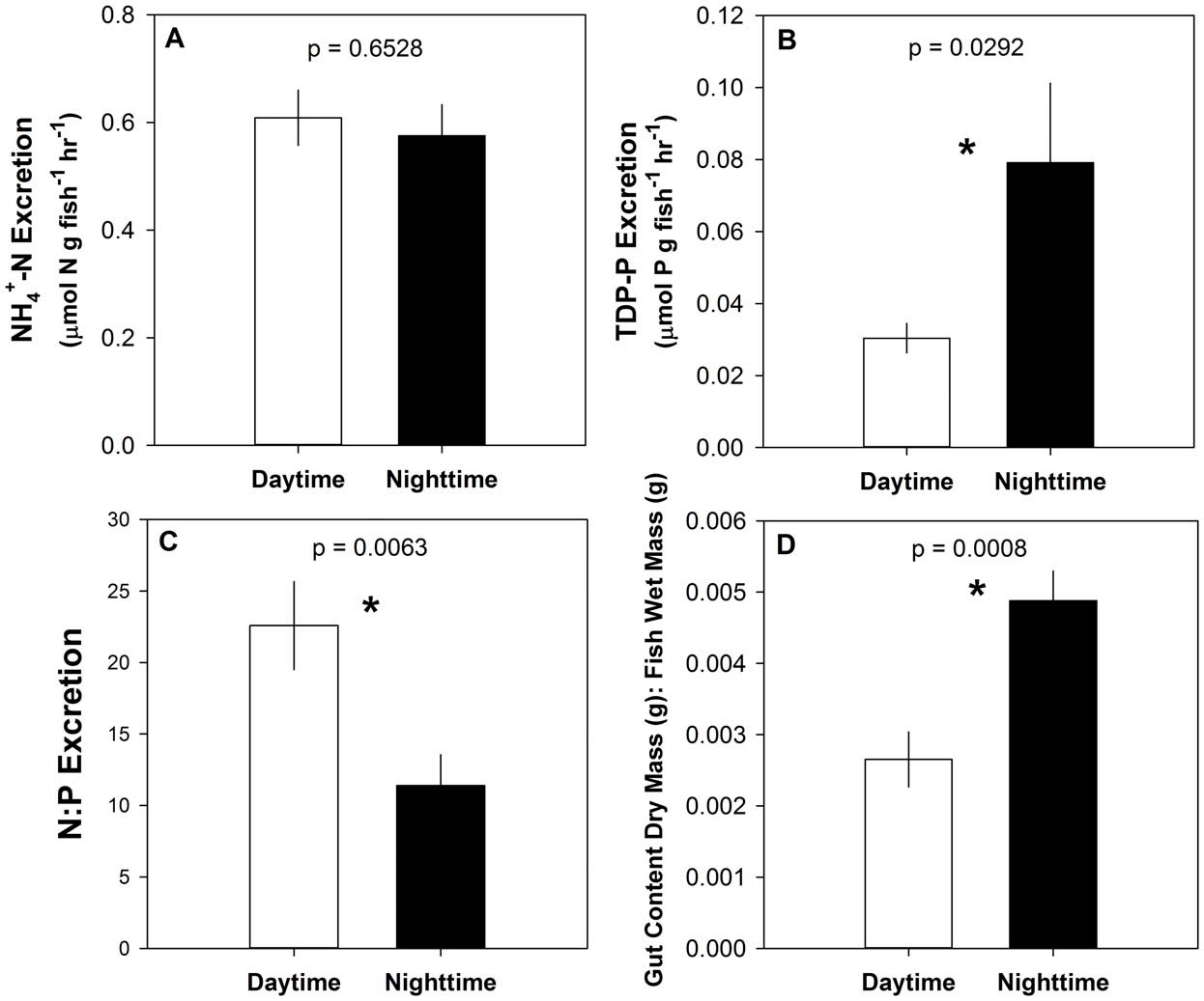
Diel changes in *Pterygoplichthys* excretion and gut content mass. Average *Pterygoplichthys* excretion and gut content mass during daytime (1000–1500 h) and nighttime hours (1900–0400 h). (A) *Pterygoplichthys* NH_4_^+^-N excretion rates; (B) *Pterygoplichthys* total dissolved phosphorus excretion rates; (C) N:P of *Pterygoplichthys* excretion; (D) gut content dry mass per wet mass of *Pterygoplichthys*. Error bars represent ±1 SE.

Average N excretion was approximately 0.6 µmol NH_4_^+^-N g wet mass ^−1 ^hr^−1^ and did not differ between the afternoon and evening sample periods (F_(1, 18)_ = 0.209, p  = 0.6528; Figure 3A). In contrast, average P excretion was twice as high in samples collected during nighttime sampling periods (approximately 0.077 µmol TDP-P g wet mass ^−1 ^hr^−1^) than those collected in the daytime (approximately 0.031 µmol TDP-P g wet mass ^−1 ^hr^−1^; F_(1, 18)_ = 5.61, p = 0.0292; Figure 3C). This resulted in a significant decrease in the N:P ratio of excretion from an average of 23 in the daytime to approximately 12 in the nighttime (F_(1, 75)_ = 9.57, p = 0.006; Figure 3C). This pattern may have been driven by nocturnal loricariid feeding behavior, evidenced by more amorphous detritus found in loricariid guts during nighttime sampling hours (F_(1, 75)_ = 12.09, p = 0.0008; Figure 3D). Loricariid size also influenced excretion rates, as larger fishes tended to excrete less N and P per gram of fish than smaller loricariids (F_(1,18)_ = 7.52, p = 0.013 and F_(1, 18) = _6.67, p = 0.019, respectively). However, loricariid size did not influence excretion stoichiometry (F_(1, 18) = _0.633, p = 0.6966). By multiplying loricariid excretion rates by their average areal biomass, we estimate that loricariids remineralize approximately 7 µmol P m ^−2 ^hr^−1^ during daylight hours, 18 µmol P m ^−2 ^hr^−1^ during nighttime hours, and 135 µmol N m ^−2 ^hr^−1^ during both time periods.

**Figure 4:**
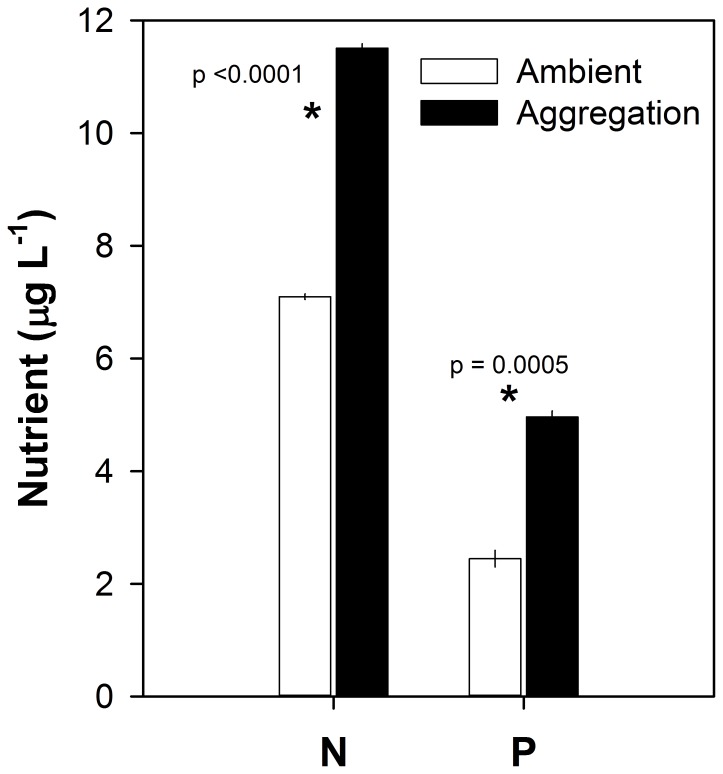
Biogeochemical hotspots created by aggregations of *Pterygoplichthys.* Means of NH_4_^+^-N and PO_4_^−3^-P (±1 SE) samples taken from paired sites within and outside of loricariid aggregations in the Chacamax River in 2008 (n = 16 ambient sites, n = 16 aggregation sites) and 2010 (n = 16 ambient sites, n = 16 aggregation sites). Aggregations were defined as water samples taken within groups of *Pterygoplichthys* that had an area of at least 5 m^2^ with at least 40 *Pterygoplichthys* per m^2^. Ambient samples were collected from sites parallel to the aggregations without immediate upstream aggregations of loricariids.

Aggregations of loricariids (Figures 1, 4) generated hotspots of nutrient recycling relative to ambient water chemistry in paired river sites. Water samples collected within aggregations of loricariids had 41% higher concentrations of NH_4_^+^-N (p<0.0001, F_(3, 60)_ = 9.63, Figure 4) and 66% higher concentrations of SRP (p = 0.0005, F_(3, 60)_ = 11.7, Figure 4) relative to paired ambient chemistry sites.

## Discussion

The results from this study support findings from other studies documenting the important role consumer nutrient recycling can play in stream ecosystems [3], [11], [33]. For example, fish excretion of the limiting nutrient N exceeded N demand in a Venezuelan stream [3]. Similarly, freshwater shrimp excretion was equivalent to approximately 20% of the N uptake and 5% of the P uptake in Puerto Rican streams [33]. In our study, excretion of N and P by high densities of loricariids appears to be a large and potentially important flux of nutrients in the Chacamax River that is both spatially and temporally heterogeneous throughout the stream reach. This is one of the first studies to measure novel biogeochemical hotspots generated by an aquarium invader.

Similar to observations reported for loricariids in their native ranges [34] and introduced populations of *Pterygoplichthys* in Florida [35], *Pterygoplichthys* in the Chacamax River were nocturnally active (Figure 2). Gut content mass was greater during evening hours, indicating loricariids are primarily active and feeding at night in the study site (Figure 3D). Loricariid behavior may have evolved to minimize predation from day-active predators, as diel changes in behavior has been attributed to predator avoidance in native populations of loricariids [36]. Unfortunately, due to the limited number of potential predators remaining in the Chacamax River and the potential large body size of *Pterygoplichthys* (up to at least 70cm SL [36]), it is unlikely that predation will control the loricariid population in the region.

For organisms to generate areas of enriched nutrient recycling rates in space, or hotspots, across a landscape, their distribution must change through space and/or time [2], [3]. In this study, we observed diurnal aggregating behavior of loricariids (Figure 1) and temporal variation in fish excretion rates (Figure 3), which created a mechanism for armored catfish to generate biogeochemical hotspots in the Chacamax River. Water samples collected within loricariid aggregations had roughly double the concentration of N and P than water collected from outside the aggregations (Figure 4), suggesting that loricariids generate localized pulses of nutrients downstream from aggregations during daylight hours when primary producers are photosynthesizing. Primary producers in the study site were nutrient limited (Figure S1) [31]. Hence, elevated ambient nutrient availability may locally alleviate some nutrient limitation and enhance algal growth and primary productivity immediately downstream of loricariid aggregations. However, loricariid aggregations disperse daily; therefore, any increase in algal biomass driven by proximity to an aggregation of loricariids is likely removed quickly by nocturnal loricariid grazing and would be difficult to detect. Future work should attempt to separate the grazing and nutrient remineralization effects of loricariids to estimate the net effects of these fishes on algal biomass and gross primary production in invaded systems.

Species-specific characteristics and areal biomass are important factors to consider when predicting if a consumer could be a significant driver of nutrient recycling or if they have the potential to increase or relax nutrient limitation [3], [12]. For example, the elemental composition, or stoichiometry, of an invader may influence the impact of changing nutrient dynamics in an ecosystem [37]. Phosphorus excretion rates are highly variable among fish species [8], [12]; thus, P-cycling in P-limited systems, such as the Chacamax River, may be strongly influenced by changes in fish communities [12]. In such a system, the introduction of a P-rich invader such as loricariids [38] may actually intensify P-limitation of primary producers and microbial heterotrophs, and influence nutrient cycling rates in streams. For example, in our site, nocturnal feeding activity (Figure 3D) may have generated increased nocturnal P excretion by loricariids (Figure 3B) and significantly altered the N:P ratio of excretion between daytime and nighttime hours (Figure 3C). Notably, the N:P ratio of excretion during daylight hours (Figure 3C) was greater than the Redfield ratio [39]; therefore, diurnal loricariid excretion may have actually enhanced P-limitation.

It is important to mention, increased loricariid activity occurred simultaneously with increases in stream water NH_4_-N concentrations, but there was no measureable change in P concentrations in the water column (Figure 2). Though we documented diel changes in P excretion by loricariids that we attributed to feeding behavior, we did not see the same pattern in N excretion (Figure 3). Most likely, increased nighttime NH_4_-N concentrations in the stream water were due to diel patterns in autotrophic ammonium demand [40] rather than differences in ammonium excretion by loricariids. Though a similar pattern may have been evident with autotrophic uptake of P, all of the P concentrations measured outside of loricariid aggregations were at or very near the level of detection. Thus, stoichiometric changes at the ecosystem level were most likely being driven by large changes in ambient ammonium concentrations. This also suggests pulses of soluble P generated by loricariid aggregations may be locally significant.

Well-mixed rivers like the Chacamax add complexity to studying the formation of biogeochemical hotspots, for unlike terrestrial environments, hotspots may not have discrete boundaries. Hence, it is important to note that loricariid remineralization contributed to the N and P concentrations of samples we collected within and outside of fish aggregations. Moreover, there was likely an array of influences loricariid invasion had on nutrient dynamics in addition to remineralization, such as bioturbation. However, we focused this study on the direct influence of invaders on nutrient cycling via remineralization. Importantly, ambient water chemistry data on the Chacamax were not collected prior to loricariid invasion, so we cannot determine the potential additive effects of loricariid nutrient recycling to total ambient solute concentrations. Moreover, P concentrations in the Chacamax were near the level of detection; thus changes in ambient P after invasion may be difficult to detect.

To be important drivers of nutrient cycling, the contribution of nutrient remineralization by organisms must be significant at the ecosystem-level [2], [3]. Our data suggest that loricariids strongly influence nutrient remineralization rates in the Chacamax River. The volumetric excretion estimates for loricariids were greater than ambient water chemistry values indicating that remineralization by loricariids may be an important flux of nutrients within the river. Moreover, loricariid biomass was two orders of magnitude greater than native fish biomass [31]. Consequently, loricariid invasion may have shifted the Chacamax River from a system where fishes were not central drivers of biogeochemical processes to a system where remineralization of nutrients by fishes is a large and important flux of nutrients.

Recent work has suggested invasive species management and eradication efforts should target species that present serious environmental risks and alter the function of ecosystems [41]. When coupled with work demonstrating non-native loricariids are: competing with native species for food resources [42], [43], altering the structure of aquatic and riparian ecosystems [44], [45], and negatively affecting populations of threatened and endangered species [35], [46]; the results from this study indicate high-densities of invading loricariids threaten the structure and function of ecosystems. Moreover, the results from this investigation provide additional evidence [47] that aggregations of non-native organisms have the potential to alter nutrient dynamics and generate biogeochemical hotspots. This work highlights the potential threat invasive, aquarium species present to ecosystem function in freshwater ecosystems.

## Supporting Information

Figure S1 **Nutrient limitation of periphyton in the Chacamax River.** Mean (±1 SE) algal biomass collected from nutrient diffusing substrates (NDS) for each of four nutrient treatments (control (CON), nitrogen (N), phosphorus (P), nitrogen and phosphorus (N+P)). Bars with different letters have significantly different (*p*<0.005) algal biomasses according to Tukey’s Honestly Significant Difference test. Nutrient diffusing substrates were constructed using methods outlined in Capps et al. [1]. They were deployed for a total of 14 days. Nutrient diffusion rate estimates were made for each nutrient treatment on day 0 and day14 by subtracting the diffusion rate of nutrient amended NDS from the rate of control NDS [1]. Nutrient diffusion rate estimates were made for each nutrient treatment by subtracting the diffusion rate of nutrient amended NDS from the rate of control NDS. On day 14 (N: 7.2×10^−5^±3.8×10^−6^; P: 7.1×10^−4^±3.1×10^−5^; N+P: (N) 9.5×10^−6^±2.1×10^−6^, (P) 8.1×10^−5^±1.5×10^−6^, mean ± SE (mol m^−2^ hr^−1^)), all treatments were diffusing less than on day 0 (N: 2.1×10^−2^±3.1×10^−4^; P: 4.9×10^−3^±4.3×10^−5^; N+P: (N) 3.9×10^−2^±3.1×10^−4^, (P) 9.1×10^−3^±5.8×10-5, mean ± SE (mol m^−2^ hr^−1^)). The results from NDS indicated that primary producers in the Chacamax River were P-limited (p<0.0001, F_(3, 43)_ = 13.6). References Cited: 1. Capps KA, Booth MT, Collins SM, Davison MA, Moslemi JM, et al. (2011) Nutrient diffusing substrata: a field comparison of commonly used methods to assess nutrient limitation. Journal of the North American Benthological Society 30∶522–532.
(TIF)

Figure S2 **Range of ventral patterns of** ***Pterygoplichthy*** **s collected in the Chacamax River (N17°29′047′′ W91°58′430′′).** *Pterygoplichthys pardalis* is characterized by a ventral pattern of dark spots (A). *Pterygoplichthys disjunctivus* is characterized by dark, vermiculated lines (G) (Armbruster & Page 2006). The wide variations in pattern suggest the *Pterygoplichthys* population in the Chacamax may be comprised of hybrids of the two species. Photo credit: K. A. Capps.
(TIF)
