# Supplementary figures and images for: Invasive Fishes Generate Biogeochemical Hotspots in a Nutrient-Limited System
Source: PLoS One. 2013 Jan 16;8(1):e54093. doi: 10.1371/journal.pone.0054093 (PMC3546933; doi:10.1371/journal.pone.0054093)

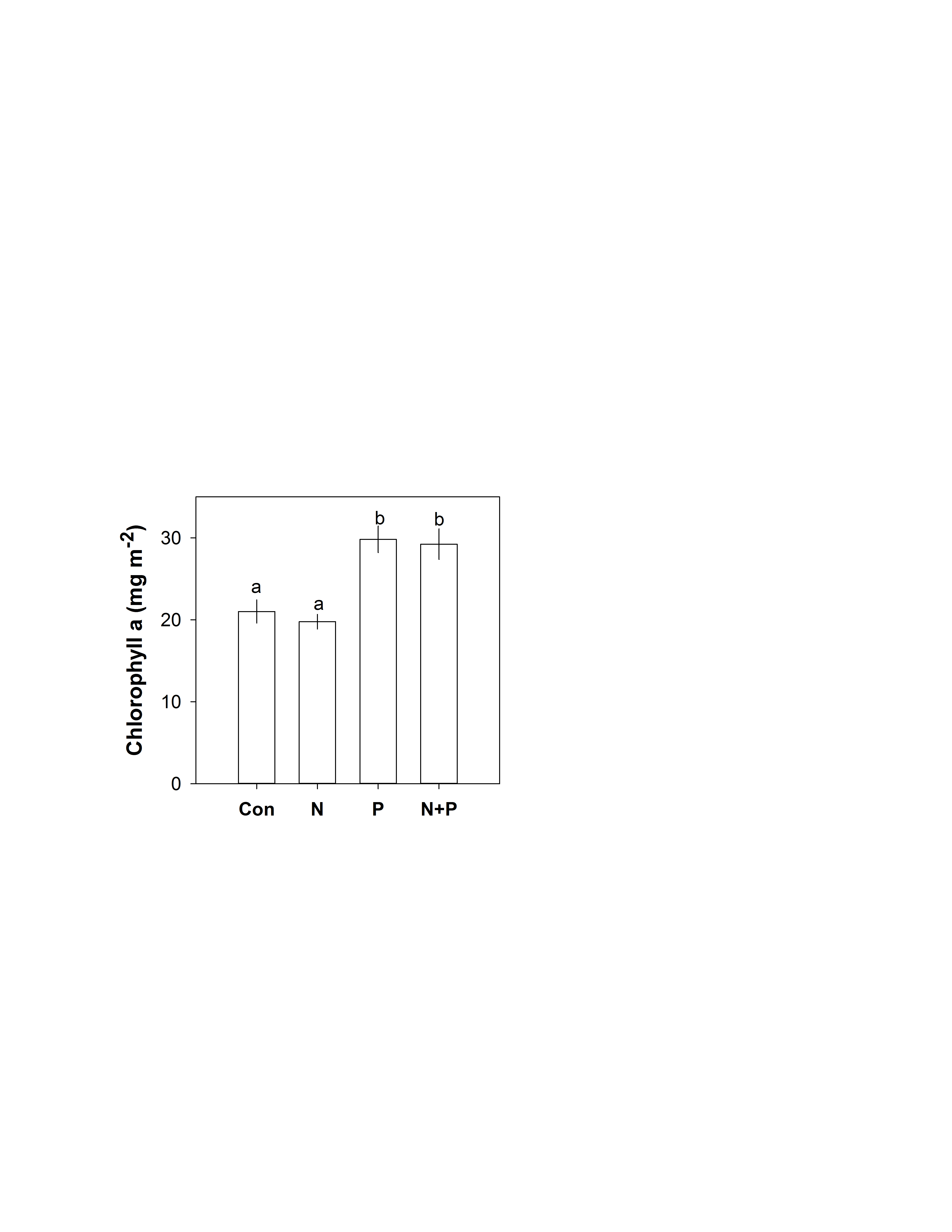

Supplement: Figure S1 — Nutrient limitation of periphyton in the Chacamax River. Mean (±1 SE) algal biomass collected from nutrient diffusing substrates (NDS) for each of four nutrient treatments (control (CON), nitrogen (N), phosphorus (P), nitrogen and phosphorus (N+P)). Bars with different letters have significantly different (p<0.005) algal biomasses according to Tukey’s Honestly Significant Difference test. Nutrient diffusing substrates were constructed using methods outlined in Capps et al. [1]. They were deployed for a total of 14 days. Nutrient diffusion rate estimates were made for each nutrient treatment on day 0 and day14 by subtracting the diffusion rate of nutrient amended NDS from the rate of control NDS [1]. Nutrient diffusion rate estimates were made for each nutrient treatment by subtracting the diffusion rate of nutrient amended NDS from the rate of control NDS. On day 14 (N: 7.2×10^−5^±3.8×10^−6^; P: 7.1×10^−4^±3.1×10^−5^; N+P: (N) 9.5×10^−6^±2.1×10^−6^, (P) 8.1×10^−5^±1.5×10^−6^, mean ± SE (mol m−2 hr−1)), all treatments were diffusing less than on day 0 (N: 2.1×10^−2^±3.1×10^−4^; P: 4.9×10^−3^±4.3×10^−5^; N+P: (N) 3.9×10^−2^±3.1×10^−4^, (P) 9.1×10^−3^±5.8×10^-5^, mean ± SE (mol m−2 hr−1)). The results from NDS indicated that primary producers in the Chacamax River were P-limited (p<0.0001, F(3, 43) = 13.6). References Cited: 1. Capps KA, Booth MT, Collins SM, Davison MA, Moslemi JM, et al. (2011) Nutrient diffusing substrata: a field comparison of commonly used methods to assess nutrient limitation. Journal of the North American Benthological Society 30∶522–532. (TIF) [file pone.0054093.s001.tif]

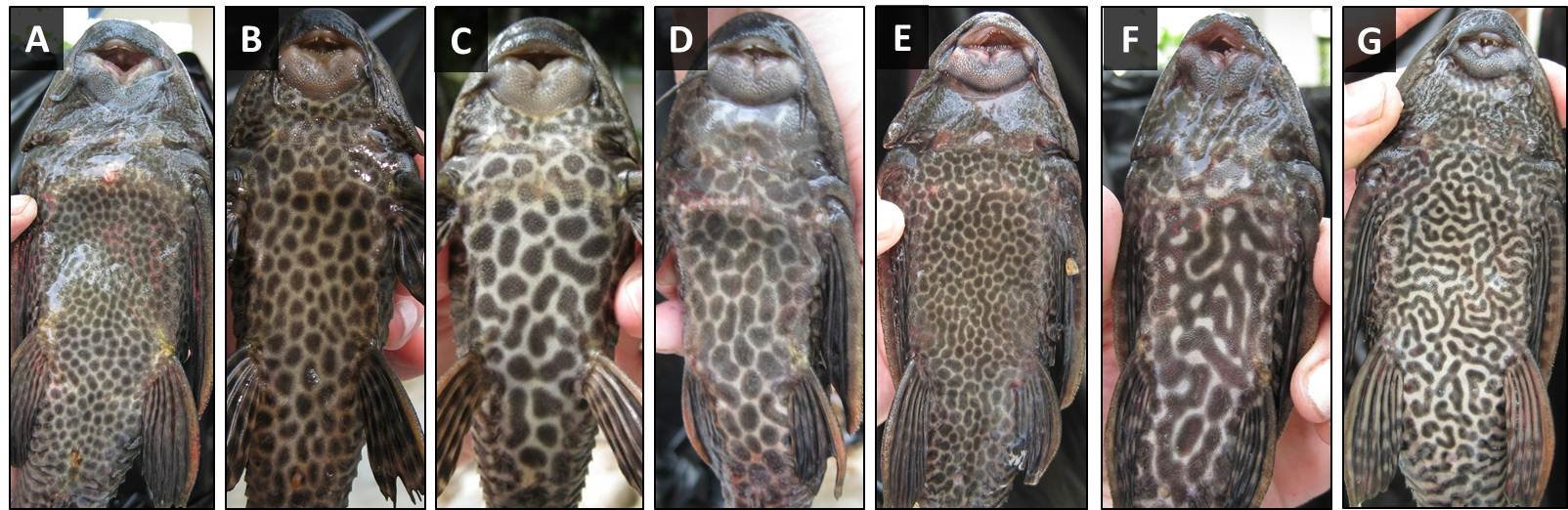

Supplement: Figure S2 — Range of ventral patterns of Pterygoplichthy s collected in the Chacamax River (N17°29′047′′ W91°58′430′′). Pterygoplichthys pardalis is characterized by a ventral pattern of dark spots (A). Pterygoplichthys disjunctivus is characterized by dark, vermiculated lines (G) (Armbruster & Page 2006). The wide variations in pattern suggest the Pterygoplichthys population in the Chacamax may be comprised of hybrids of the two species. Photo credit: K. A. Capps. (TIF) [file pone.0054093.s002.tif]
